# Supplementary material for: Engineered Nanoparticles with Decoupled Photocatalysis and Wettability for Membrane-Based Desalination and Separation of Oil-Saline Water Mixtures
Source: Nanomaterials (Basel). 2021 May 25;11(6):1397. doi: 10.3390/nano11061397 (PMC8227411; doi:10.3390/nano11061397)
Supplement: Supplementary file 1 [file nanomaterials-11-01397-s001.zip › nanomaterials-1233520-supplementary.pdf]

nanomaterials

MDPI

Supporting Information

Engineered Nanoparticles with Decoupled Photocatalysis and Wettability for Membrane-Based Desalination and Separation of Oil-Saline Water Mixtures

Bishwash Shrestha [†], Mohammadamin Ezazi [†] and Gibum Kwon [*]

Department of Mechanical Engineering, University of Kansas, Lawrence, KS 66045, USA; bishwashs@ku.edu (B.S.); aminezazi@ku.edu (M.E.)

* Correspondence: gbkwon@ku.edu

† Equal contributions.

1. The Size Distribution and Transmission Electron Microscopy of N-TiO_2 Nanoparticles

We determined the size distributions of nitrogen-doped TiO_2 (N-TiO_2) nanoparticles by utilizing the dynamic light scattering (DLS) method (See also **Section 2** in the main text). The DLS data show that the average size of N-TiO_2 nanoparticles is 50 nm ± 1 nm (**Figure S1**a). Further, the transmission electron microscopy (TEM) images show that N-TiO_2 nanoparticles are highly crystalline with an average lattice spacing of ≈ 0.35 nm which corresponds to (101) plane of anatase TiO_2 (**Figure S1**b and **S1**c).

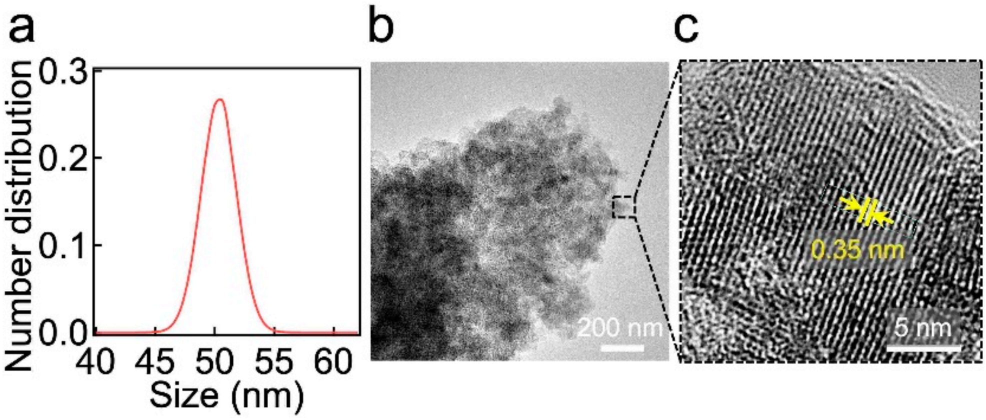

Figure S1. (a) A number size distribution of N-TiO_2 nanoparticles obtained by utilizing the DLS method. **(b,c)** Transmission electron microscopy (TEM) image of N-TiO_2 nanoparticles (**b**) and high-resolution TEM image showing N-TiO_2 lattice structure with an average spacing of ≈ 0.35 nm (**c**).

2. X-ray Diffraction Patterns of N-TiO_2 Nanoparticles

X-ray diffraction (XRD) patterns were utilized to determine the crystal structure of N-TiO_2 nanoparticles (**Figure S2**). The characteristic peaks at 2θ = 25.27°, 37.94°, 48.16°, 54.07°, 55.12°, 62.73°, 69.06°, 70.65°, and 75.33° correspond to the anatase phase with lattice planes (101), (103), (200), (105), (211), (204), (116), (220), and (107), respectively. Nitrogen doping resulted in a slight decrease in the intensity of these peaks (i.e., broadening). This can be attributed to the alteration in the crystallite size [1]. Note that doping with nitrogen did not cause any phase transformation.

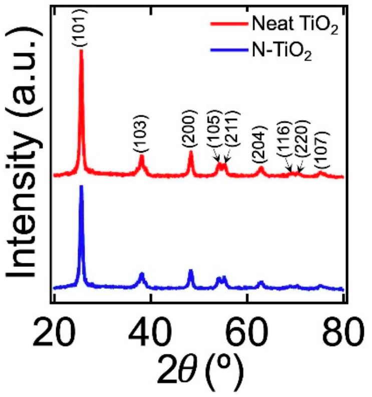

Figure S2. XRD pattern of N-TiO_2 synthesized by using a molar ratio of TEA and TBOT as 2.0. The XRD pattern of a neat TiO_2 is also shown.

3. The Effect of the Visible Light Intensity on the Photocatalytic Degradation of Dye Molecules

We studied the effect of the visible light intensity on the photocatalytic degradation of organic dye molecules on N-TiO_2 nanoparticles. **Figure S3** demonstrates the UV-Vis absorption spectra of water solutions dissolved with Solvent Blue 38 dye (concentration = 0.5 wt%) and N-TiO_2 nanoparticles (concentration = 0.5 wt%) after 2h of visible light irradiation with the intensity of ≈ 198, ≈ 399, and ≈ 598 mW cm^{-2}. The results show that the dye molecules were degraded more rapidly upon irradiation of a higher intensity light. We also verified that the decrease in the dye concentration is mainly due to photocatalytic degradation by N-TiO_2 nanoparticles instead of other non-catalytic origins such as adsorption. We conducted the same test for 10 hours in dark (i.e., no visible light irradiation). The results showed an insignificant decrease in the intensity of the absorption spectra indicating that the dye was barely degraded in dark.

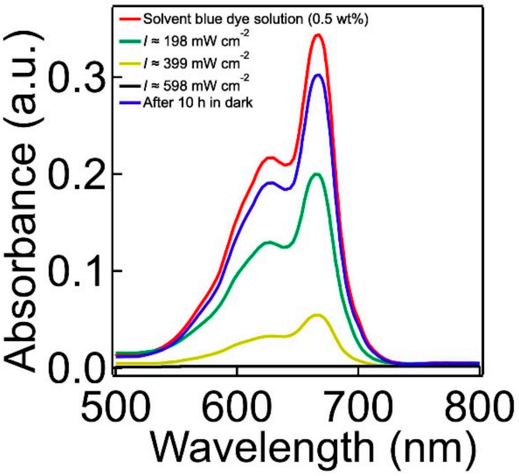

Figure S3. UV-Vis absorption spectra of water solutions dissolved with Solvent Blue 38 dye (concentration = 0.5 wt%) and N-TiO_2 (concentration = 0.5 wt%) after 2h of visible light irradiation with varied intensity. The UV-Vis absorption spectra of water solution dissolved with Solvent Blue 38 dye (concentration = 0.5 wt%) and N-TiO_2 (concentration = 0.5 wt%) after 10h in dark is also provided.

4. The Synthesis and Size Distribution of F-SiO_2 Nanoparticles

Figure S4a demonstrates the synthesis of silica nanoparticles with low surface energy (F-SiO_2) by hydrolysis of tetraethyl orthosilicate (TEOS) followed by grafting of 1H,1H,2H,2H-perfluorodecyl trichlorosilane (i.e., perfluoro silane) (See also **Section 2** in the main text). We determined the size distributions of F-SiO_2 nanoparticles by utilizing

the DLS method (see **Section 2** in the main text). The DLS data revealed that the average size of F-SiO_2 nanoparticles is 50 nm ± 2 nm (**Figure S4**b). It is worth noting that the average size of F-SiO_2 nanoparticles is very close to that of N-TiO_2 nanoparticles (See **Figure S1**a). This is critical to ensure that the N-TiO_2/F-SiO_2 mixture forms a homogeneous and uniform coating on the membrane surface.

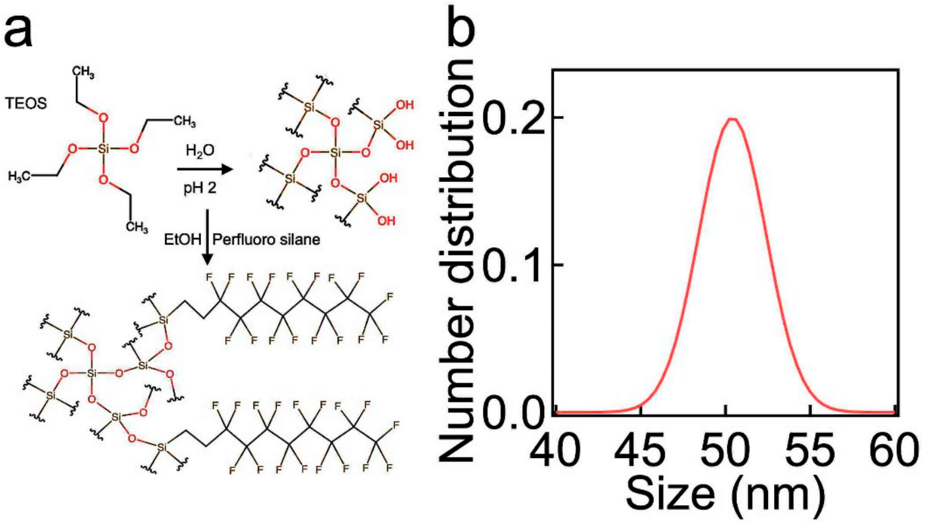

Figure S4. **(a)** Schematic illustrating the synthesis of F-SiO_2 nanoparticles by utilizing tetraethyl orthosilicate (TEOS) as SiO_2 precursor and 1H,1H,2H,2H-perfluorodecyl trichlorosilane (i.e., perfluoro silane). **(b)** A number size distribution of F-SiO_2 nanoparticles obtained by utilizing the DLS method.

5. The Relations between the Water Contact Angle and the Breakthrough Pressure

We and others demonstrated that a membrane's breakthrough pressure (i.e., the minimum pressure difference across the membrane that is required for a liquid to permeate through) for water decreases with a decrease in the contact angle for water [2]. Therefore, such a membrane exhibits a higher flux for water-rich permeate. Here we measured the breakthrough pressure for saline water (1.0 wt% NaCl in DI water) through the membranes coated with N-TiO_2/F-SiO_2 with varied compositions (**Figure S5**). The results show that a membrane possessing a higher N-TiO_2 composition exhibits a lower breakthrough pressure for saline water. Note that a membrane coated with a higher N-TiO_2 composition in N-TiO_2/F-SiO_2 mixture exhibited a lower saline water contact angle (See **Figure 3**b in the main text).

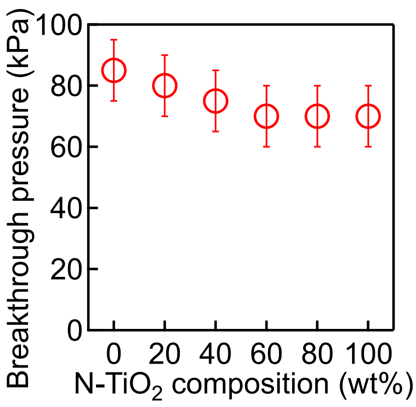

Figure S5. A plot of the measured breakthrough pressure for saline water (1.0 wt% NaCl in DI water) of the membranes coated with N-TiO_2/F-SiO_2 with varied compositions.

6. Determining the Salt Rejection by Measuring the Electrical Conductivity.

We determined the salt (NaCl) concentration in the water-rich permeate by calculating the electrical conductivity of the permeate and compared the value with the calibration curve. Two probes of a multimeter (Gardner Bender GDT-3190) at a distance of 2 cm were submerged in the water solution. The multimeter measures the electrical resistance (R). The electrical resistivity (r) can be calculated by utilizing the equation, $R = rL/A$, where L and A are the distance between two probes (i.e., 2 cm), and the surface area of the probe (i.e., 20 mm^2) submerged in the water solution. Subsequently, the inverse of electrical resistivity yields the electrical conductivity (s) of the water solution (i.e., $s = 1/r$). **Figure S6**a presents the calibration curve of the electrical conductivity of DI water as a function of NaCl concentrations.

We then measured the salt rejection (ξ) of the membranes coated with various concentrations of N-TiO_2/F-SiO_2 (**Figure S6**b). Here, we define the salt rejection as $\xi = 1 - \sigma_f/\sigma_o$, where σ_o and σ_f are the concentrations of salt in the feed of saline water (i.e., 1.0 wt%) and those in the permeate obtained in t = 180 minutes, respectively. By comparing the ξ values for the membranes coated with various compositions of N-TiO_2/F-SiO_2 with those of the as-purchased commercial membrane or those coated only with cured adhesive, we found that the N-TiO_2/F-SiO_2 had a negligible effect on salt rejection.

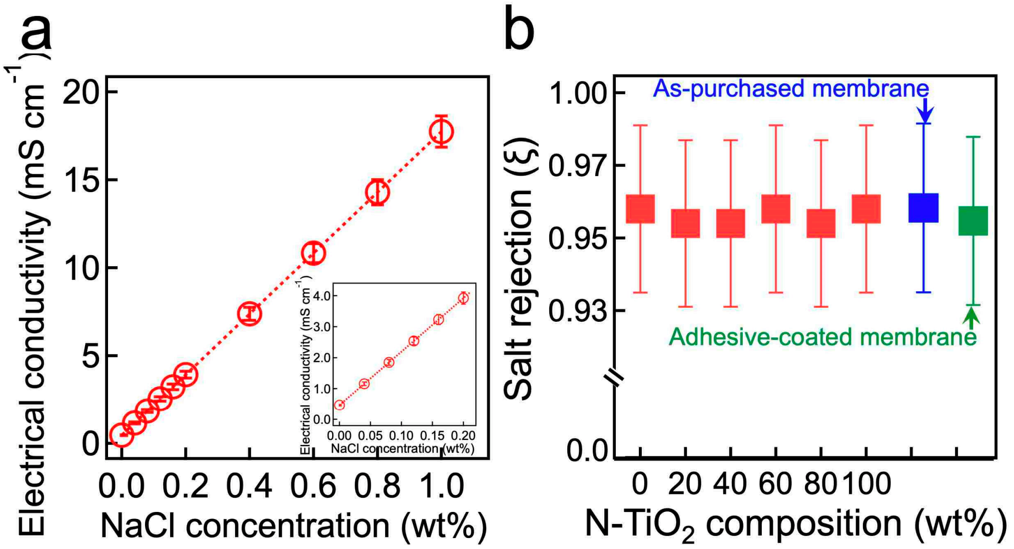

Figure S6. **(a)** The calibration curve established by calculating the electrical conductivity of water as a function of salt (NaCl) concentrations. Inset shows zoomed-in electrical conductivity data in the NaCl concentration range of 0.00-0.20 wt%. **(b)** The salt rejection (ξ) data of the membranes coated with N-TiO_2/F-SiO_2 with varied compositions. The data of the as-purchased commercial membrane and that coated only with cured adhesive are also provided for comparison.

7. The Adhesion of N-TiO_2/F-SiO_2 to the Membrane Surface

High shear force by the feed stream exerted on the membranes often causes the delamination of the coating from the membrane surface. We evaluated the adhesion of N-TiO_2/F-SiO_2 to the commercial membrane surface by measuring the mass of the membranes after 180 minutes of continuous desalination of saline water (1.0 wt% NaCl in DI water) at a flow rate of 1.5 L min^{-1} and compared the values with that of the as-prepared membranes. Here we utilized a high-precision balance (Mettler Toledo XS105 DU, precision = 0.010 ± 0.002 mg). The results show that the mass of the as-prepared membranes and those of the membranes after the test remained nearly unchanged (i.e., the mass loss < 0.1%, **Figure S7**). Here we define the mass loss (m_L) as $m_L = (1 - m_f/m_o) \times 100$, where m_o and m_f are the initial mass of N-TiO_2/F-SiO_2 mixture coated to the membrane, and that after subjecting the membrane to saline water desalination for 180 minutes, respectively. This

can be attributed to the cured adhesive which can hold N-TiO_2 and F-SiO_2 nanoparticles together and securely bind them to the membrane surface by forming an interlocking structure between the nanoparticles and the membrane. In contrast, the membranes prepared without cured adhesive exhibited ≈ 89 ± 2% mass loss.

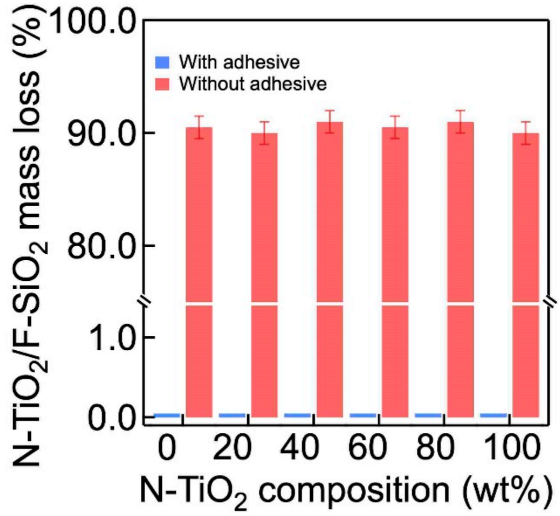

Figure S7. The mass loss of the membranes coated with N-TiO_2/F-SiO_2 with varied compositions after 180 minutes of desalination of saline water at a flow rate of 1.5 L min^{-1}. For comparison, the results obtained by utilizing the membranes without cured adhesive are also shown.

8. Determining the Oil Concentration in the Water-rich Permeate

Although our membranes are fouled by oil during the separation of the oil-saline water mixture, we showed that the oil concentration in the water-rich permeate remained very low. We determined the oil concentration by utilizing thermogravimetric analyses (TGA, PerkinElmer PYRIS 1). Approximately 10 mg of the water-rich permeate was heated from room temperature (≈ 22 °C) to 110 °C at a rate of 5 °C min^{-1} followed by maintaining at 110 °C for 50 minutes. Given that the boiling points of water and oil (n-hexadecane) are 100 °C and ≈287 °C [3], respectively, the sample remained after TGA can be assumed as pure oil. **Figure S8** shows the TGA data of the water-rich permeate through the membranes coated with N-TiO_2/F-SiO_2 with varied compositions. The results indicate that the concentrations of oil in the water-rich permeates are very low (i.e., < 0.1 wt%). This can be attributed to the membranes' extremely low permeability.

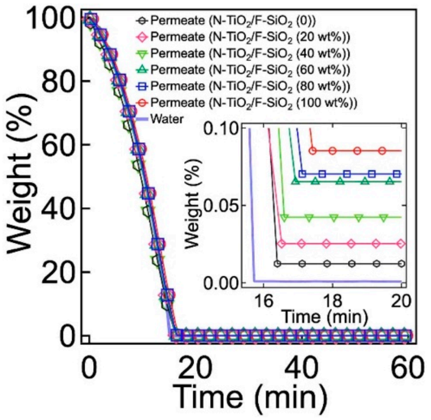

Figure S8. The TGA data of the water-rich permeates after the separation of SDS-stabilized oil-in-water emulsion dissolved with NaCl (1.0 wt% with respect to water mass) through the membranes coated N-TiO_2/F-SiO_2 with varied compositions.

9. Effect of Photocatalytic Degradation Reaction on the F-SiO_2.

Our membranes exhibited unchanged contact angles for oil (n-hexadecane) after 120 minutes of visible-light-driven photocatalysis (See **Figure 4**d in the main text). We attribute this to the fact that the membrane's surface chemistry remained unchanged. **Figure S9** presents the FT-IR spectra of a membrane's surface coated with N-TiO_2/F-SiO_2 (60 wt%) after 120 minutes of visible light irradiation ($I \approx 198$ mW cm^{-2}). By comparing it with that of the as-prepared membrane, we can verify that the surface chemistry remains unaffected. Particularly, the absorption peaks in the range of ≈1100 cm^{-1} to ≈1400 cm^{-1} which correspond to C-F in perfluoro silane remained nearly unchanged.

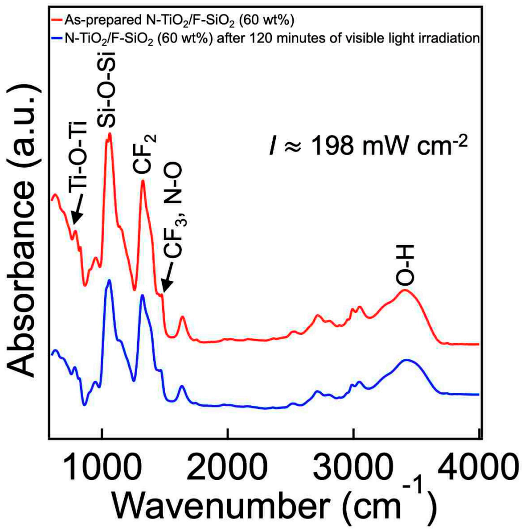

Figure S9. The FT-IR spectra of a membrane surface coated with N-TiO_2/F-SiO_2 (60 wt%) after 120 minutes of visible light irradiation with an intensity of ≈198 mW cm^{-2}. For comparison, the FT-IR spectra of the as-prepared membrane surface is shown.

10. The Cured Adhesive Layer and Salt Rejection after Visible-light-driven Photocatalysis

We verified that the cured adhesive (Norland Optical Adhesive 61) remains unaffected after visible-light-driven photocatalysis on the membrane surface. We conducted FT-IR measurements by utilizing PerkinElmer Spectrum 400 FT-IR Spectrometer in attenuated total reflectance (ATR) mode. The FT-IR spectra were recorded at a resolution of 4 cm^{-1} for 16 scans. A sample was prepared by mixing adhesive and N-TiO_2 with a 1:1 weight ratio in acetone (solute concentration = 1.0 wt%) followed by casting the mixture on a polytetrafluoroethylene (PTFE) substrate. Then, the film was cured by UV light (λ = 365 nm, $I \approx 78$ mW cm^{-2}) for 5 minutes at room temperature (≈ 22 °C). Finally, the film was detached from the substrate and ground for FT-IR measurements. The survey spectra of the adhesive after 180 minutes of visible light irradiation ($I \approx 198$ mW cm^{-2}) are shown in **Figure S10**. Note that the spectra of neat N-TiO_2 were set as the background. By comparing the survey spectra with that of the as-prepared adhesive, we found that the absorption peaks underwent negligible alterations after visible light-driven photocatalysis.

The salt rejection (ξ) of the membranes coated with various concentrations of N-TiO_2/F-SiO_2 was measured after 60 minutes of visible light irradiation. The salt rejection remained almost constant (**Figure S11**). We attribute this to the cured adhesive layer which protected the commercial membrane's active layer (e.g., polyamide) from a reactive radical species that was generated from exposure to visible light irradiation [4].

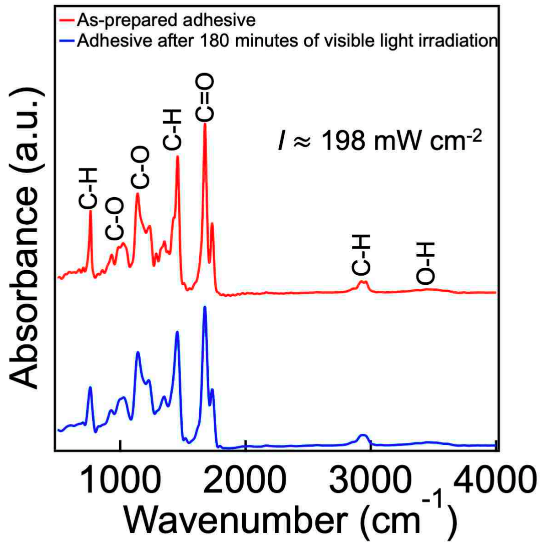

Figure S10. The FT-IR spectra of the cured adhesive after 180 minutes of visible light-driven photocatalysis by N-TiO_2 ($I \approx 198$ mW cm^{-2}). For comparison, the FT-IR spectra of the as-prepared adhesive is also shown.

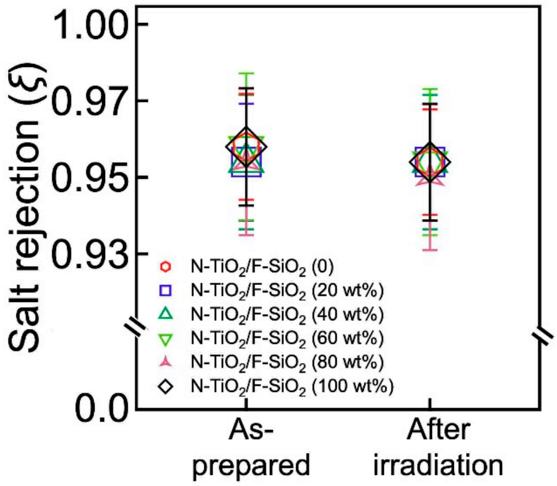

Figure S11. The salt rejection (ξ) of the membranes coated with N-TiO_2/F-SiO_2 with varied compositions after exposure to 60 minutes of visible light irradiation.

Movie Legends

Supporting Movie S1. This movie demonstrates that water displaces an oil droplet from the coated membrane.

References

1. Chen, X. and C. Burda, The electronic origin of the visible-light absorption properties of C-, N-and S-doped TiO2 nanomaterials, *Journal of the American Chemical Society* **2008**, *130,* 5018–5019.
2. Lafuma, A. and D. Quéré, Superhydrophobic states, *Nature materials* **2003**, *2*, 457–460.
3. Camin, D.L., A.F. Forziati, and F.D. Rossini, Physical properties of n-hexadecane, n-decylcyclopentane, n-decylcyclohexane, 1-hexadecene and n-decylbenzene, *The Journal of Physical Chemistry* **1954**, *58*, 440–442.
4. Bartolo, D., G. Degré, P. Nghe, and V. Studer, Microfluidic stickers, *Lab on a Chip* **2008**, *8*, 274–279.
